# Multiscale assessment of treatment efficacy in adults with ADHD: A randomized placebo-controlled, multi-centre study with extended-release methylphenidate

**DOI:** 10.3109/15622975.2010.540257

**Published:** 2010-12-14

**Authors:** Wolfgang Retz, Michael Rösler, Claudia Ose, André Scherag, Barbara Alm, Alexandra Philipsen, Roland Fischer, Richard Ammer, The Study Group

**Affiliations:** 1 Neurocentre, Saarland University Hospital, University of the Saarland, Homburg, Germany; 2 Institute for Medical Informatics, Biometry and Epidemiology, University Essen, Essen, Germany; 3 Central Institute of Mental Health, Mannheim, Germany; 4 Department of Psychiatry and Psychotherapy, University Medical Center, Freiburg, Germany; 5 Medice, Arzneimittel Pütter GmbH & Co. KG, Iserlohn, Germany

**Keywords:** Adult ADHD, methylphenidate, stimulants, treatment, randomized clinical trial

## Abstract

**Objectives:**

This trial was performed to test the efficacy and safety of an extended-release formulation of methylphenidate (MPH ER).

**Methods:**

A total of 162 adults with ADHD according to DSM-IV were treated for 8 weeks with either two daily individually body weight-adjusted doses of MPH ER up to 1 mg/kg per day (*N* = 84) or placebo (*N* = 78). The primary efficacy outcome was the Wender-Reimherr Adult Attention Deficit Disorder Scale (WRAADDS) 8 weeks after randomization. Secondary efficacy measures were the ADHD Diagnostic Checklist (ADHD-DC), the Conners Adult Attention Deficit Disorder Scale (CAARS-S:L), the Clinical Global Impression (CGI) and the Sheehan Disability Scale (SDS).

**Results:**

At week 8 a significantly higher decline of the total WRAADDS score was found in the MPH ER group as compared to the placebo group (*P* = 0.0003). The rates of responders were 50% in the MPH ER and 18% in the placebo group (*P* < 0.0001). Furthermore, similar effects were observed for the secondary efficacy variable: ADHD-DC score (*P* = 0.004), CAARS-S:L score (*P* = 0.008) and the SDS score (*P* = 0.017). 50% of the MPH ER group and 24.4% of the placebo group were improved “much” or “very much” according to the CGI rating (*P* = 0.0001). MPH ER treatment was well tolerated. At week 2 also the mean heart rate was significantly higher in the MPH ER group as compared to the placebo group (*P* = 0.01). No differences between the study groups were observed regarding mean blood pressure at any visit.

**Conclusions:**

This clinical trial demonstrated statistically significant and clinical relevant effects of MPH ER in adults with ADHD for several self- and investigator-rated ADHD psychopathology and also functional efficacy measures.

## Introduction

Attention-deficit/hyperactivity disorder (ADHD) is one of the most common psychiatric conditions in childhood, with approximately 6–9% of school-age children being affected (Barkley and Murphy 1998). Studies have found that up to 60–65% of affected children continue to suffer from this disease as adults, with it becoming a chronic condition in approximately 3.4% adult people worldwide (Fayyad et al. 2007). Considering the prevalence of adult ADHD and the negative impact its symptoms may have on the different domains of a patient's life (Goodman 2007), it should be recognized as an important mental disorder requiring accurate identification and treatment (Rösler et al. 2010a).

The longest-used medications for ADHD are stimulants which have been used in the management of childhood ADHD since the 1930s (Bradley 1937). Methylphenidate was first synthesized 1944, and introduced into therapeutics in 1954. Beginning in the 1960s, it was used to treat children with ADHD. Until the 2000s, options in terms of stimulants were limited to immediate-release (IR) and first-generation extended-release (ER) formulations (Stein 2004). Now, a number of extended-release stimulant medications are available in European and non-European countries (Rösler et al. 2010a).

Based on their favorable efficacy profiles, stimulants have become the standard medical treatment for ADHD in children and adults and are recommended as first line pharmacological treatment as one component of multimodal therapy by several evidence-based guidelines (Ebert et al. 2003; Nutt et al. 2007; NICE 2008). Stimulants have been proved effective in the treatment of ADHD in all ages. A growing number of clinical studies has given substantial evidence that MPH treatment is efficacious and safe also in adult ADHD patients. In several meta-analyses moderate to high standardized effect sizes (Cohen's *d*') on the reduction of ADHD core symptoms were calculated from studies of appropriate design and methodological standards. In a first meta-analysis, Faraone et al. (2004) included six trials with methylphenidate in adult ADHD. They found a mean effect size of *d*’ = 0.9 and no evidence of publication bias. Larger MPH effect sizes were associated with physician ratings of outcome and use of higher doses. More recently, an overall effect size of *d*’ = 0.42 was reported from a meta-analysis of 16 studies with methylphenidate in adult ADHD patients (Koesters et al. 2009). Regression analysis showed no significant influence of mean daily dose on effect size and no publication bias. Efficacy of pharmacological treatment of 1991 adults with ADHD from 11 stimulant and non-stimulant treatment trials has been analyzed in a third meta-analytical approach by Mészáros et al. (2009). The effect size for stimulants was *d*’ = 0.67 and somewhat higher than for non-stimulant medications, where an effect size of *d*’ = 0.59 was calculated from the data. Similarly, in a meta-analysis of 19 treatment studies a significant higher effect size was found for stimulants (*d*’ = 0.73 and 0.86 for long-acting and short-acting stimulants, respectively) than for non-stimulants (*d*’ = 0.39) (Faraone and Glatt 2010).

Beside the reduction of ADHD symptoms according to DSM-IV, which focus on inattention, hyperactivity and impulsivity, recent pharmacological trials have also considered treatment effects on additional symptom domains which are related to ADHD in adult patients like disorganization and problems with emotional regulation. Decline of different measures of emotional dysregulation, oppositional symptoms and disorganization in adults with ADHD have been shown as a result of methylphenidate treatment in several randomized placebo-controlled trials (Reimherr et al. 2007; Rösler et al. 2009, 2010b; Marchant et al. 2010). Also improvement of self concept and action control has been observed under methylphenidate treatment in adults with ADHD (Edel et al. 2009). Moreover, positive effects of stimulants on a functional level and measures of quality of life have been reported (Spencer et al. 2008). Likewise, a significant effect of methylphenidate on social adjustment over a period of one year could be demonstrated (Wender et al. 2010). Thus, there is much evidence from a large number of independent studies that stimulant medications and methylphenidate in particular have a beneficial effect on ADHD core and associated symptoms, and also on every day functioning.

The purpose of this study was to test the hypotheses that treatment with an extended release formulation of methylphenidate (MPH ER) not only reduces (1) the core symptom domains of ADHD, inattention, hyperactivity and impulsiveness, but also (2) the broader psychopathological spectrum of adult ADHD according to the Utah criteria, which comprise symptoms of emotional dysregulation and disorganization, and (3) may improve every day functioning in adults with ADHD. In this study we used a twice a day dosing schedule with a sustained release MPH preparation instead of a single dose in the morning in order to adapt treatment effects to the needs of adult ADHD patients. Moreover, this study was the first in adults with ADHD that employed individually body-weight adjusted daily MPH ER doses for an optimal balance of efficacy and side effects.

## Methods

Subjects were outpatients with ADHD aged 18 years and older. For study inclusion the subject had to fulfil the DSM-IV criteria for ADHD (314.00 and 314.01). The diagnosis was established by clinical assessment and by use of a German standardized diagnostic instrument for psychiatric experts (ADHD-DC, Rösler et al. 2004). A retrospective assessment of DSM-IV ADHD symptoms was made in the presence of an informant whenever possible. In addition, the German short version of the Wender Utah Rating scale (WURS, Wender 1995) was administered to all subjects in order to make sure that childhood ADHD symptoms were present by a retrospective self report of the patient. A cut-off score of at least 30 points served as an indicator of apparent childhood ADHD psychopathology (Retz-Junginger et al. 2002, 2003). Subjects with WURS-k scores <30 were not included.

Before inclusion of a subject in the study, each case has been re-evaluated by a central control committee of experienced psychiatrists (MR, WR) for verification of patient eligibility. Anamnestic data, standardized ADHD ratings and diagnostic interviews (WRAADDS, SKID-I/-II), were cross-checked for any inconstancies in order to make the diagnoses of ADHD as sure as possible. Inclusion and exclusion criteria were carefully re-examined to avoid protocol violations.

The German versions of the SCID-I and -II interviews (SKID-I and -II, Wittchen et al. 1997) were used to assess comorbid psychiatric diagnoses. Individuals with low intelligence (IQ < 85), dementia, schizophrenia, bipolar disorder, current major depression, acute anxiety disorders and other unstable psychiatric conditions were excluded, as were subjects with any serious medical illness. Also subjects with drug or alcohol dependence during the 6 months before screening, pregnant or nursing women, persons with a Body Mass Index <20 or body weight ≥130 kg, and individuals treated with any psychopharmacological drug in addition to study medication were not included. Urine screenings for drugs of abuse were performed at screening visit and at week 8 and could be repeated at any time of the study at the investigator's discretion. A wash-out period of at least 2 weeks was necessary for any psychopharmacological drug before study inclusion.

The study was approved by the ethical committee of the State of the Saarland and has been performed in accordance with the ethical standards laid down in the 1964 Declaration of Helsinki. The trial has been registered with the Federal Opium Agency at the Federal Institute for Drugs and Medical Devices as well as at ClinicalTrials.gov (NCT00730249). Subjects were included in the study after provided written informed consent.

### Procedure

A double-blind, randomized, placebo-controlled study with parallel-group design was conducted at 10 sites. The treatment period was 8 weeks with a 2-week titration and a 6-week maintenance phase.

Randomisation was performed by Medice's Galenic Department which included the generation of the randomisation list and the preparation of emergency envelopes. We used block randomisation with a block size of 4. The block size was not mentioned in the investigational plan or the consent given to patients.

MPH ER is a MPH preparation manufactured by Medice Company (Germany) with a proportion of 50% immediate release MPH and 50% of extended release MPH. The effective time of action is 7–8 h (Garcia-Garcia et al. 2009). The effects of a single dose of this drug are comparable to the effects of twice daily immediate release MPH. The drug is available in several European countries and has been approved for the treatment of children and adolescents with ADHD up to age 18 years. Its dosing profile predicts a larger immediate and smaller delayed action than OROS MPH, with individual differences in time course being observed. The drug was described and compared with other long acting MPH medications by the European guideline group (Banaschewski et al. 2006).

Medication was individually titrated b.i.d. after breakfast and lunch during the first 2 weeks to an optimal dose on the basis of tolerability and according to the body weight with a maximum daily dose of approximately 1 mg/kg body weight, starting with 10–30 mg/day. Patients were assigned to one of four weight classes (less than 55 kg, 55–69 kg, 70–104 kg and 105–130 kg) with doses of 40, 60, 80 and 120 mg daily, respectively. The interval between the two doses was 6–8 h. A standardised disease management programme consisting of seven sessions was administered individually to all participants of the study. The programme has already been used in an earlier study (Rösler et al. 2009) and was implemented in this study as a mandatory part of the treatment according to advice of the German authorities, to improve compliance and to avoid inconsistencies regarding information about and coping with ADHD within the study population. Disease management sessions were performed at baseline and six visits up to week 8. During these sessions patients received information about ADHD aetiology and symptoms, support in perception of symptoms and specific problems, help with the management of self-regulation and emotional problems, time management and performing daily routines.

### Assessments

Each subject underwent a comprehensive clinical assessment by a certified psychiatrist using standardized rating scales and interviews. The clinical examination included medical history, physical and neurological examination, assessments of vital parameters, body weight, liver function tests, complete blood count, ECG and EEG.

The primary outcome measure was the total score of the Wender-Reimherr Adult Attention Deficit Disorder Scale (WRAADDS, Wender 1995, Rösler et al. 2010c). The WRAADDS has been validated in German populations by Rösler et al. (2008a,b). This structured interview consists of 28 items in seven psychopathological domains, which are rated by a clinical expert on a 0–2 Likert scale. The psychopathological domains are inattention, hyperactivity, affective lability, hot temper, stress intolerance, disorganization and impulsivity. The raters were required to attend training with observed interviews to standardize rating practices for this instrument before the study started. WRAADDS assessments were performed at screening and at the end of the double-blind maintenance phase (week 8). Subjects were required to have a WRAADDS score of more than 35 points at screening to be included into the study.

Assessments of the ADHD DSM-IV criteria at screening and at week 8 were performed by use of the German version of the ADHD Rating Scale-IV (ADHD RS-IV, DuPaul et al. 1998), which is the ADHD-DC (Rösler et al. 2004). This expert rating refers to the nine psychopathological DSM-IV criteria for inattentiveness and the nine criteria for hyperactivity and impulsivity. Each item is rated by an expert as present (1) or not present (0), resulting in a maximum score of 26.

The 66-item long version of the CAARS self report scale (CAARS-S:L, Conners et al. 1999) was used as a second outcome measure. This instrument comprises the 18 DSM-IV items of ADHD psychopathology. The CAARS-S:L was administered at screening and at week 8. An inconsistency index >7 at baseline lead to exclusion from the study.

Overall severity, change of severity, improvement, overall therapeutic effects and tolerability were assessed with the Clinical Global Impression scale (CGI, NIMH 1985). CGI ratings were performed at screening and at week 8.

The Sheehan Disability Scale (SDS) was used for the assessment of the degree to which ADHD interferes in function regarding three domains of daily living: work/school, social and family life. The extent of impairment in these domains was rated by the patient on a 0–10 point visual analogue scale. The three items were summed up to a single measure of global functional impairment that ranges from 0 to 30 (Sheehan 1983, Sheehan and Sheehan 2008). SDS scores were assessed at screening and at week 8.

Adverse events were assessed at each visit. Patients were asked for any complaints and new adverse symptoms. In addition, standardized assessments of adverse events were performed at weeks 1, 2 and 8 by use of the somatic symptom sheet of the AMDP-system (AMDP 2006; Guy and Ban 1982). This rating scale contains 40 items referring to 10 neurological and 30 somatic symptoms.

### Statistical analyses

The total score of the WRAADDS after 8 weeks was analysed as primary variable. The total score is the sum of all items (in case of missing items the sum was divided by the number of items answered and multiplied by 28). Missing data were imputed using the last-observation carried forward (LOCF) procedure (only one measurement was missing).

The primary analysis was performed on the intent-to-treat (ITT) population using a linear mixed effects model with fixed effect treatment, covariate baseline WRAADDS and random effect centre. We applied a significance level of α = 0.05 (two-sided) for the primary confirmatory test (*F*-test of the fixed effect). Additional sensitivity analyses were performed to test the robustness of the primary analyses with regard to the imputed data (under a best- and a worst-case scenario). All secondary endpoints were analysed exploratively either with the Wilcoxon [*U*-test (at least ordinal scale) or with Fisher's exact test (binary data).

A sample size of 64 in each treatment group has 80% power to detect a difference in means of 7 assuming that the common standard deviation is 14 under a two-group *t*-test scenario with a significance level α of 5% (two-sided). Since a dropout rate of about 15% was expected, a total of 75 patients should be recruited for each of the two treatment groups.

## Results

**Figure 1:**
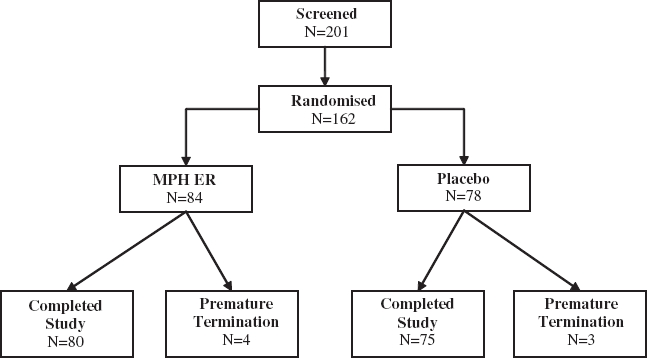
Flow diagram of subject progress.

A total of 162 patients were randomized and included in this trial. 84 patients received MPH ER and 78 placebo during the double-blind study period (Figure 1). Seven subjects discontinued the study prematurely, four in the placebo group and three in the MPH ER group. Reasons for premature discontinuation were adverse events in three patients of the MPH ER group and one of the placebo group. One patient in the MPH ER group discontinued due to lack of efficacy. In the placebo group one drop-out was related to the need of antihypertensive medication, another withdrew the consent.

Prior to randomization, i.e. at the beginning of the treatment phase, we also compared both groups descriptively. There was no evidence for difference in mean age, ADHD symptom score by ADHD-DC and WRAADDS score, CGI severity ratings, SDS score and IQ between the MPH ER and the placebo group at the beginning of the treatment phase. Some evidence for differences was found regarding sex and body weight/BMI with a preponderance of women and subjects with lower body weight/BMI in the MPH ER group (Table I). The mean CAARS-S:L score was also higher in the MPH ER group than in the placebo group at the beginning of the treatment phase. The incidence of comorbid conditions according to SKID-I interviews is shown in Table II. No evidence for difference was observed for the proportion of individuals who had received earlier MPH treatment (29.8% in the MPH ER group and 37.2% in the placebo group; Fisher's exact test, *P* = 0.32).

**Table I:** Demographic and clinical characteristics of the intent-to-treat study population at screening. Data are presented as N (%) or mean ± SD.

	MPH ER *N* = 84	Placebo *N* = 78	Statistics *P* values
Age (years)	36.6 ± 10.4	38.2 ± 9.9	Wilcoxon *U*-test
			*P* = 0.42
Sex			Fisher's exact test
Male	32 (38%)	44 (56%)	*P* = 0.0272
Female	52 (61%)	34 (44%)	
Body weight (kg)	73.8 ± 13.7	82.9 ± 17.1	Wilcoxon *U*-test
Male	80.7 ± 11.2	89.2 ± 17.1	*P* = 0.0005
Female	69.6 ± 13.4	74.7 ± 13.5	
IQ	112.2 ± 13.4	113.3 ± 14.3	Wilcoxon *U*-test
			*P* = 0.67
ADHD-DC score			Wilcoxon *U*-test
Inattention	7.8 ± 1.0	7.6 ± 1.0	*P* = 0.24
Hyperactivity/Impulsivity	7.1 ± 1.1	7.1 ± 1.0	*P* = 0.99
WRAADDS score	46.3 ± 5.0	45.4 ± 5.3	Wilcoxon *U*-test
			*P* = 0.3471
CAARS-S:L score	126.1 ± 31.7	114.4 ± 30.4	Wilcoxon *U*-test
			*P* = 0.0230
CGI Severity of illness score	5.2 ± 0.7	5.2 ± 0.7	Wilcoxon *U*-test
			*P* = 0.81
SDS score	19.7 ± 4.7	19.0 ± 5.5	Wilcoxon *U*-test
			*P* = 0.55

**Table II:** Probable and confirmed DSM-IV axis-1 and axis-II diagnoses according to SCID-I and -II interviews of the intent-to-treat study population (*N*).

	MPH ER, *N* = 84	Placebo, *N* = 78
DSM-IV Axis-I	lifetime/current	lifetime/current
Bipolar disorders	0/0	0/0
Major depression	16/0	15/0
Depression NOS	4/0	3/1
Dysthymia	0/2	0/3
Affective disorder caused by specific factor	0/0	1/0
Substance-induced depressive disorder	0/0	0/0
Psychotic disorders	0/0	0/0
Alcohol abuse/dependence	3/0	9/0
Drug abuse/dependence*	7/0	11/0
Panic disorder	1/0	2/0
Phobic disorders*	17/7	12/5
Generalized anxiety disorder	1/0	1/0
Anxiety disorder caused by specific factor	0/0	0/0
Substance-induced anxiety disorder	0/0	0/0
Anxiety disorder NOS	1/1	0/0
Obsessive-compulsive disorder	4/2	3/0
PTSD	0/0	2/0
Somatization disorder/hypochondriasis*	n.a./2	n.a./4
Body dysmorphic disorder	n.a./1	n.a./0
Eating disorders*	5/0	7/1
Adjustment disorders	n.a./1	n.a./0
Others	3/1	3/0
DSM-IV Axis-II		
Avoidant	20	11
Dependent	6	5
Obsessive-compulsive	30	29
Negativistic	18	12
Depressive	12	11
Paranoid	13	7
Schizotypic	1	2
Schizoid	4	2
Histrionic	1	3
Narcissistic	6	9
Borderline	16	14
Antisocial	8	7
Personality disorder NOS	0	0

* Multiple meanings per patient possible.

At week 8 the mean daily doses at week 8 were 66 ± 20 mg in the MPH ER group (males 80.0 ± 8.8 mg, females 66.9 ± 15.8 mg) and 78 ± 17 mg in the placebo group (Wilcoxon *U*-test, *P* = 0.0001). These are equivalent to 0.9 ± 0.2 mg/kg body weight MPH ER and 0.9 ± 0.1 mg/kg body weight placebo, respectively (Wilcoxon *U*-test, *P* = 0.2091). Note that there were slightly more women in the MPH-ER group and as women weigh on average less than men this resulted in a lower average daily dose in milligrams in the MPH ER group. In addition, dose reductions were descriptively more frequent in the MPH-ER than in the placebo group (19 vs. 2 patients). There was no evidence for group differences regarding compliance (96% in the MPH ER group and 97% in the placebo group).

### Efficacy

**Figure 2:**
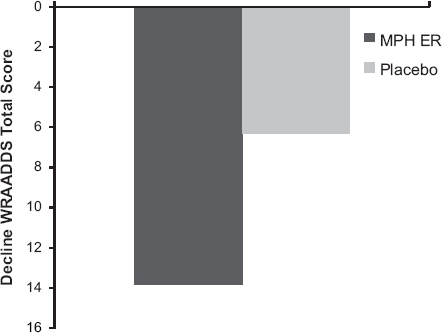
Mean reduction of the WRAADDS total score at week 8 by treatment group (ITT population, Wilcoxon [*U*-test, *P* = 0.0003).

There was a marked decline of 13.8 points on the WRAADDS in the MPH ER group as compared to 6.2 points in the placebo group during the 8 week double-blind treatment period (Figure 2). At week 8, the WRAADDS score was 32.5 (95% confidence interval (CI) 29.7–35.3) in the MPH ER group and 39.2 (95% CI 36.6–41.8) in the placebo group. The mean difference between MPH ER and placebo was 6.8 points (95% CI 3.2–10.4) which can be translated into a standardized effect size of 0.54 in units of pooled standard deviations (Cohen's *d*'). The difference between the MPH ER group and the placebo group at week 8 was statistically significant (ITT population, confirmatory analysis, mixed linear model, *P* = 0.0003). The results were confirmed by robustness analyses with worst- and best-case imputations (*P* = 0.0002 and *P* = 0.0011, respectively) and also by analysis of the per protocol (PP) population, which consisted of 143 patients who completed the study without any major protocol violations (mixed linear model, *P* = 0.0003). The effect size in this analysis was *d*’ = 0.59.

Explorative analyses of the WRAADDS subscales revealed substantial differences at week 8 between patients of the MPH ER and the placebo group with the exception of the subscales hot temper and emotional over-reactivity. The highest effect size of *d*’ = 0.68 was found regarding attention difficulties (Wilcoxon *U*-test, *P* = 0.00004). Marked effects were also found regarding hyperactivity and impulsivity (Wilcoxon *U*-test, *P* = 0.00129 and *P* = 0.00447, respectively), which refer together with attention difficulties to the core symptoms of ADHD according to the concept of DSM-IV. The effect sizes were *d*’ = 0.51 and *d*’ = 0.46, respectively. Evidence for effects were also found regarding disorganisation (*d*’ = 0.42; Wilcoxon *U*-test, *P* = 0.00715) and affective lability (*d*’ = 0.31; Wilcoxon *U*-test, *P* = 0.04698).

Similar differences were found for other secondary measures: At week 8, patients on the placebo and the MPH ER group also differed regarding ADHD-DC symptom scores (*d*’ = 0.54; Wilcoxon *U*-test, *P* = 0.004). The decline on the ADHD-DC was 5.6 points in the MPH ER group and 2.6 points in the placebo group. Differences between placebo and MPH ER patients at week 8 were found for both symptom domains – inattention (*d*’ = 0.49; Wilcoxon *U*-test, *P* = 0.009) and hyperactivity/impulsivity (*d*’ = 0.58, Wilcoxon *U*-test, *P* = 0.0009).

**Figure 3:**
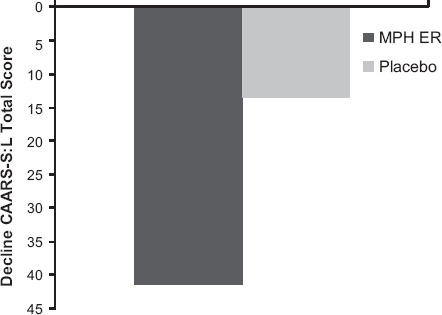
Mean reduction of the CAARRS-S:L total score at week 8 by treatment group (ITT population, Wilcoxon *U*-test, *P* = 0.008).

In the MPH ER group the decrease of the CAARS-S:L total score was 41.5 points over the 8-week treatment period. In the placebo group the decline was 13.1 points (Figure 3). Note that the placebo group showed a slightly higher mean CAARS-S:L score as compared to the MPH ER group at the beginning of treatment (Wilcoxon *U*-test, *P* = 0.023). However, we observed evidence for a difference between patients treated with MPH and those treated with placebo at week 8 for the secondary outcome CAARS-S:L total score (*d*’ = 0.44; Wilcoxon *U*-test, *P* = 0.008).

**Figure 4:**
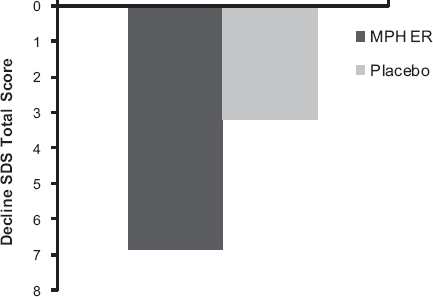
Mean reduction of the SDS total score at week 8 by treatment group (ITT population, Wilcoxon [*U*-test, *P* = 0.017).

Finally, we also observed evidence for a difference regarding functional improvement on the SDS at week 8 between the MPH ER and the placebo group (Wilcoxon *U*-test, *P* = 0.0167). The total score decline was 6.9 points in the MPH ER group and 3.1 points in the placebo group (Figure 4; *d*’ = 0.40).

The Spearman correlation coefficient between the total scores of self and investigator ratings in the entire patient group at week 8 were 0.72 for ADHD-DC and CAARS-S:L and 0.76 for WRAADDS and CAARS-S:L (both *P* < 0.0001). The Spearman correlation coefficients between the expert ratings ADHD-DC and WRAADDS was 0.81 (*P* < 0.0001). Regarding the SDS total score, Spearman correlation coefficients were 0.67 for the correlation with the WRAADDS total score, 0.57 for the correlation with the ADHD-DC total score, and 0.72 for the correlation with the CAARS-S:L total score (all *P* < 0.0001).

### Responder analyses

**Figure 5:**
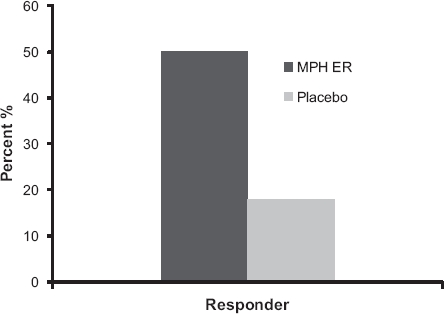
Responders according to reduction of the WRAADDS total score by 30% in the MPH ER and the placebo group.

The number of responders according to the criterion of a 30% reduction of the WRAADDS total score at study endpoint was 50% for the MPH ER group and 18% for the placebo group (Figure 5; Fisher's exact test, *P* < 0.0001).

Moreover, the improvement assessed by expert clinical global impression (CGI) also indicated between-group differences. 50% of the MPH ER patients and 24.3% of the placebo group showed “very much” or “much” improvement at week 8 (Wilcoxon *U*-test, *P* = 0.0001). Marked and moderate therapeutic effects were seen in 50% of the patients treated with MPH and in 24.4% of those treated with placebo (Wilcoxon *U*-test, *P* = 0.0001).

Because of the inhomogeneous sex distribution between the MPH ER and placebo group, the analyses of the primary and secondary outcomes were additionally stratified by sex. Overall, the results for WRAADDS, CAARS-S:L, CGI, ADHD-CL and the Sheehan Scale all favoured the general claims for MPH ER in subgroups of males and females.

### Adverse events

In the double-blind treatment phase 151 adverse events were reported in 55 patients of the MPH ER group, and 77 in 32 patients of the placebo group (Fisher's Exact Test, *P* = 0.003). 63% of all adverse events were rated as “moderate". One serious adverse event was observed in the MPH ER group (rupture of the acromioclavicular joint), and two were reported in one patient of the placebo group (appendicitis and postoperative pain). The relation between SAEs and study medication were rated as “unlikely” or “not related".

Reduced appetite, dry mouth and increased thirst were the most frequent side effects assessed with the AMDP adverse events list. These side effects were more frequently reported for the MPH ER group as compared to the placebo group (Table III). Backache, excessive appetite and seborrhoea were more frequently reported under placebo. Most adverse events were recorded at the end of the titration phase at week 2.

**Table III:** Adverse events measured by somatic symptom scale of AMDP. Only those adverse events are listed, which were significantly more frequent in either treatment group.

MPH ER > Placebo	Max. difference at week	MPH ER (%)	Placebo (%)
Decreased appetite	W2	48	10
Dry mouth	W2	38	14
Excessive thirst	W2	32	9
Headache	W1	30	17
Palpitations	W8	25	6
Dizziness	W2	23	9
Gastric discomfort	W2	17	5
Nausea	W1	17	4

Placebo > MPH ER		Placebo (%)	MPH ER (%)

Backache	W1	24	10
Excessive appetite	W8	19	4
Drowsiness	W2	19	8
Seborrhoea	W2	8	1

### Vital signs

There was a small but clinically not meaningful increase of mean systolic and diastolic blood pressure during the treatment period in both treatment groups (Table IV). No significant differences were seen between the treatment groups at any visit. The mean heart rate in the MPH ER group showed an increase compared to placebo at week 2 (*P* = 0.01). The difference compared to placebo was 4 bpm. At week 8 there was still some descriptive evidence for a difference between the MPH ER and the placebo group (76 vs. 73 bpm; *P* = 0.08).

**Table IV:** Week by week pulse (bpm), systolic and diastolic blood pressure (BP) values. *P* values are given for treatment group differences.

		MPH ER	Placebo	Wilcoxon *U*-test *P* values
Screening	Systolic BP	121 ± 11	124 ± 11	0.08
	Diastolic BP	79 ± 8	81 ± 6	0.51
	Pulse	71 ± 10	71 ± 9	0.80
Baseline	Systolic BP	121 ± 13	124 ± 11	0.14
	Diastolic BP	79 + 9	81 ± 7	0.16
	Pulse	73 ± 11	75 ± 9	0.17
Week 1	Systolic BP	123 ± 12	126 ± 14	0.12
	Diastolic BP	80 ± 9	81 ± 9	0.61
	Pulse	78 ± 11	74 ± 10	0.08
Week 2	Systolic BP	124 ± 11	125 ± 12	0.58
	Diastolic BP	81 ± 10	81 ± 10	0.86
	Pulse	78 ± 11	74 ± 10	0.01
Week 8	Systolic BP	123 ± 12	125 ± 12	0.61
	Diastolic BP	80 ± 9	83 ± 8	0.14
	Pulse	76 ± 11	73 ± 11	0.08

## Conclusions

In this randomized clinical trial statistically and clinically relevant effects of MPH ER in adult patients with ADHD were found in comparison to placebo. Differences between patients treated with MPH ER and placebo for 8 weeks were observed for both the primary and the secondary outcomes, i.e. regarding standardized measures of expert ratings (WRAADDS, ADHD-DC) as well as on the patients’ self-reports (CAARS-S:L) of ADHD symptoms, the global clinical situation (CGI) and everyday functioning (SDS). Thus, the results of this study provide evidence for improvement of psychopathology and functional impairment associated with ADHD from the therapists’ and the patients’ perspective, respectively.

The findings of this study also suggest that MPH ER treatment results not only in relevant amelioration of ADHD core symptoms, which comprise symptoms of inattention, hyperactivity and impulsiv-ity according to DSM-IV, but also of ADHD according to the Utah-criteria for adult ADHD. This concept has been introduced by P.H. Wender (1995) prior to the introduction of the attention disorders by DSM-III (1980) and goes beyond DSM-IV. It comprises a broader psychopathological spectrum and includes the domains disorganization, problems with temper control, affective lability and emotional over-reactivity beside the classical triade of inattention, hyperactivity and impulsivity. It is now widely accepted that emotional dysregulation and disorganization in daily activities are present as associated symptoms in the majority of adult cases with ADHD (Gibbins and Weiss 2007). The high correlation between the WRAADDS and ADHD-DC ratings, which refer to the Utah- and DSM-IV criteria respectively, found in this study indicates that both concepts are closely related and that, therefore, emotional dysregulation and disorganization might present intrinsic ADHD psychopathology.

The WRAADDS has been used in several clinical trials with adults with ADHD (Michelson et al. 2003; Reimherr et al. 2005, 2007; Marchant et al. 2010). The results from this study confirm findings from other clinical trials that suggest amelioration of ADHD core and related symptoms by MPH (Reimherr et al. 2007; Marchant et al. 2010; Rösler et al. 2010b), particularly of disorganization and affective lability. The treatment effect of MPH ER on the primary outcome WRAADDS total score proved to be robust as statistical significance was confirmed by worst- and best-case imputations and also by analysis of the study population without violations of the study protocol (per protocol population).

It is another important finding of this study that MPH treatment has not only a beneficial effect on ADHD symptoms from the expert's view, but also from the patients themselves. We found a decline of the mean CAARS-S:L total score of about one third in the MPH group during the 8 weeks of treatment. This adds additional support to the clinical relevance of the results of this study. In this context it is notable that also the global clinical situation, rated with the CGI and also functional disabilities regarding work/school, social and family life, which have assessed with the SDS, were improved by MPH treatment. We found moderate to high correlations between ADHD self and expert ratings and functional impairment assessed with the SDS, which might suggest that social problems and ADHD are no independent phenomena. Above all, the finding of functional improvement together with clinical symptoms of ADHD underlines the clinical relevance of MPH ER treatment effects found in this study. Thus, our results are in line with other recent studies regarding the efficacy of stimulants in adult ADHD, which showed improvement of executive functions, quality of life and social adjustment (Spencer et al. 2008; Weiss et al. 2010; Wender et al. 2010). Taken together, there is converging evidence from this and other studies that MPH improves psychopathological and functional problems in ADHD patients.

The effect sizes (Cohen's *d')* found in this study ranged between 0.31 regarding amelioration of affective lability and 0.68 regarding the improvement of attention difficulties. The effect size on the primary outcome measure, the WRAADDS total score was 0.54 in the intent-to-treat and 0.59 in the per protocol population, respectively. The effect size on the primary outcome measure in this study compares to those seen in earlier MPH studies in adult ADHD and exceeds those found in large efficacy studies, e.g., of antidepressants in affective disorders (Bech et al. 2000). The effect sizes, which have been calculated from ratings with several instruments, are in the range of those from recent meta-analyses of pharmacological treatment in adult ADHD. They are somewhat lower than those reported by Faraone et al. (2004, 2009) and Mészáros et al. (2009) for stimulants (0.67–0.9), but higher than the mean effect size of 0.42, which has been reported by Koesters et al. (2009). In an earlier study with MPH ER and a study design very similar to the recent study, an effect size of *d’* = 0.39 on the WRAADDS as the primary efficacy parameter was found (Rösler et al. 2009). In this previous clinical trial much lower doses of MPH ER and no individual body weight adjusted titration of study medication were used. The mean MPH ER dose in that earlier study was 0.55 mg/kg body weight and 0.9 mg/kg body weight in the present study. Thus, it might be concluded that the therapeutic effect of MPH ER depends on the daily dose and that higher doses are associated with higher efficacy.

Although the mean daily doses used in this study were not far from the recommended upper dose limit of 1.0 mg/kg body weight per day, treatment with MPH ER was well tolerated and the observed side effects like reduced appetite and dry mouth resembled those reported from other clinical studies. Moreover, most side effects were observed during the titration phase and were moderate and short-lasting. In contrast to prior short-term investigations with long-acting MPH formulations (Biederman et al. 2006; Reimherr et al. 2007), we found no significant effect on blood pressure in this study. A slight mean elevation of the heart rate was observed during titration in the MPH ER treatment when compared to the placebo group. As a consequence, monitoring of vital signs has to be recommended when adults are treated with MPH ER.

Some limitations of this study have to be mentioned. The first point refers to the diagnostic process in ADHD patients, as diagnosing ADHD correctly is a crucial issue in any study with adults suffering from this disorder. In this study we used standardized instruments for the diagnostic procedure, which were applied by well trained psychiatrists. Moreover, each case has been re-evaluated by a central control committee before randomization of the subject. Nevertheless, it might be a limitation that – in contrast to ADHD studies with children and adolescents – assessments of ADHD symptoms and diagnoses were mainly based on self-reports of the subjects. In particular, also assessment of childhood ADHD symptoms did not necessarily rely on third person observations in this study. However, diagnostic interviews which were primarily used in this study are the fundamental basis of every diagnostic process in adult psychiatry and it has been shown that adults present competent sources of information and that self-reports may lead to valid diagnoses in subjects with ADHD in particular (Murphy and Schachar 2000; Rösler et al. 2006).

Another limitation of this study might be the implementation of a basic disease management program with psychoeducational elements. This program was already used in a further study (Rösler et al. 2009) according to advices of the German authorities. As knowledge about ADHD and the competences of coping with ADHD symptoms are very variable across adults with ADHD, we decided to run this basic psychosocial intervention in order to minimize inconsistencies within the study population. Thus, it cannot be excluded that some therapeutic effects observed in this trial might be due to this additional non-pharmacological intervention. However, as all patients of the MPH ER and the placebo group were provided with the same program and the placebo responder rate was similar or lower than in other recent trials in adults with ADHD (Biederman et al. 2006; Rösler et al. 2009), no major non-pharmacological treatment effect can be assumed.

In conclusion this study showed both statistically significant and clinically relevant improvements in ADHD symptoms based on several expert ratings and self-report instruments. Further, treatment with MPH ER was associated with a decline of ADHD symptoms according to DSM-IV as well as according to the broader concept of the Utah criteria. Importantly, also functional impairment of patients was improved after 8 weeks of treatment with MPH ER. Treatment with body weight adjusted mean daily MPH ER doses of 0.9 mg/kg was well tolerated and no severe adverse events related to study medication were observed during the study. Although no clinical relevant changes of vital signs were observed after 8 weeks of treatment, some small drug effects on the heart rate during the titration phase were observed. Therefore, controls of vital signs have to be recommended in the treatment of adults with MPH.

## Statement of interest

WR has received honoraria and travel grants from Novartis, AstraZeneca, Medice and Flynn Pharma and is on the advisory board of Shire. MR has received honoraria, travel grants, and paid consultancy from Lilly, Janssen-Cilag, Shire and Medice and is on the advisory board of Lilly and Shire. CO has received honoraria from Medice. AS currently serves as trial statistician is on a data safety and monitoring the advisory board for Boehringer Ingelheim KG. BA is on the advisory board of Lilly. AP has received honoraria and travel grants from Janssen-Cilag, Medice, Lilly, Novartis and is on the advisory board of Janssen-Cilag, Lilly and Shire. RF is the Medical Director of Medice. RA is member of the general management of Medice and sponsor of the trial.
